# Expression of PD-L1 and p-RPS6 in epithelial dysplasia and squamous cell carcinoma of the oral cavity

**DOI:** 10.3389/froh.2024.1337582

**Published:** 2024-02-02

**Authors:** Jaruwat Hanroongsri, Panomwat Amornphimoltham, Rania H. Younis, Risa Chaisuparat

**Affiliations:** ^1^ Division of Oral Diagnostic Sciences, Faculty of Dentistry, Thammasat University, Pathumthani, Thailand; ^2^ Department of Oral Biology, Faculty of Dentistry, Mahidol University, Bangkok, Thailand; ^3^ Department of Oral Pathology, Faculty of Dentistry, Alexandria University, Alexandria, Egypt; ^4^ Department of Oral Pathology, Faculty of Dentistry, Chulalongkorn University, Bangkok, Thailand; ^5^ Avatar Biotechnologies for Oral Heath and Healthy Longevity, Faculty of Dentistry, Chulalongkorn University, Bangkok, Thailand

**Keywords:** oral epithelial dysplasia, oral squamous cell carcinoma, PD-L1, RPS6, malignant transformation

## Abstract

**Introduction:**

Oral squamous cell carcinoma (OSCC) is often preceded by oral epithelial dysplasia (OED). The role of ribosomal protein S6 (RPS6) and programmed cell death ligand-1 (PD-L1) in the progression of OED to OSCC remains unclear. This study aimed to investigate the expression of phosphorylated RPS6 (p-RPS6) and PD-L1 in OSCC and OED and to examine its relationship with clinicopathological features.

**Methods:**

Fifty-two OSCC and 48 OED cases were recruited for immunohistochemical analysis of p-RPS6 and PD-L1 expression. The expression of markers was correlated with clinicopathological features of OSCC and OED.

**Results:**

We found p-RPS6 expression in all cases of OSCC and OED, whereas PD-L1 was expressed in 42/48 (87%) OED and in 28/52 (53%) OSCC. The patients with mild OED presented higher expression level of PD-L1 and p-RPS6 significantly, when compared to moderate-differentiated OSCC patients (*p* < 0.05). Moreover, we found a significant positive correlation between PD-L1 and p-RPS6 expression in OED and OSCC patients (*p* < 0.01). The PD-L1 expression was significantly related to more than 2 cm tumor size in OSCC patients (*p *=* *0.007).

**Discussion:**

Our findings suggest the upregulation of PD-L1 may be related with activation of the mTOR pathway in the early events of tumor progression and the pathogenesis of OSCC.

## Introduction

Head and neck cancer is among the top ten leading cancers worldwide (1). The lip and oral cavity are the most affected sites for head and neck cancer (1, 2). Oral squamous cell carcinoma (OSCC) is the most common of oral cancer (1). A subset of the OSCC is proceeded by oral potentially malignant disorders (OPMDs). The World Health Organization (WHO) (3) defines OPMDs as “clinical presentations that carry a risk of cancer development in the oral cavity, whether in a clinically definable precursor lesion or in clinically normal oral mucosa.” These disorders include leukoplakia, erythroplakia, erythroleukoplakia, oral submucous fibrosis, palatal lesion of reverse cigar smoking and oral lichen planus. Oral epithelial dysplasia (OED) is one of the histopathological features of OPMDs, characterized by loss of uniformity of individual epithelial cells and their architectural orientation. Dysplastic changes include pleomorphism, hyperchromatic nuclei, abundant mitotic figures, and loss of progressive maturation of basal cells (3). OED is a spectrum of architectural and cytological epithelial changes caused by an accumulation of genetic alterations associated with the progression to OSCC (3). A systematic review and meta-analysis in 2015 (4) showed that the malignant transformation rate (MTR) in 3,708 patients presenting with OED was approximately 10.5%. Moreover, severe OED showed a higher MTR than the other OED levels.

In recent years, advances in molecular biology have been applied to study carcinogenesis signaling pathways. The phosphatidylinositol 3-kinase (PI3K), Akt, and mammalian target of rapamycin (mTOR) pathways are related to diverse cellular processes, including protein synthesis, cell proliferation, and cell growth (5–7). The mTOR regulates protein synthesis through activation of ribosomal protein S6 (RPS6) and inactivation of eukaryotic translation initiation Factor 4E-binding protein 1 (4E-BP1) (5, 8). Various cancers have reported dysregulation of mTOR pathway components (9) and the overexpression of RPS6 and 4E-BP-1 is associated with malignant progression. Accordingly, the accumulation of phosphorylated form of the RPS6, a typical downstream product of the mTOR pathway is one of the most frequent events in head and neck squamous cell carcinoma (HNSCC) especially in OSCC (8, 10).

In addition to conventional studies of biomarkers on tumor cells, new insights have emphasized the role of the immune microenvironment. The immunosuppressive process between tumor cells and host immune cells may be related to the ability of dysplastic or tumor cells to escape immune attack (11). Programmed death-ligand 1 (PD-L1) is a checkpoint inhibitor that can be triggered by interferon-gamma (IFNγ), and the receptor for PD-L1 is programmed cell death protein 1 (PD-1) (12). This receptor is predominantly released from activated CD8+ T cells in the tumor microenvironment. PD-L1/PD-1 interaction interrupts immune surveillance and promotes tumor progression through various mechanisms. One of the important mechanisms is the overexpression of PD-L1 on cancer cells, which prevents tumor cell apoptosis from inhibiting anti-tumor immune responses (13, 14). In HNSCC, the expression of PD-L1 in cancer cells is related to effector T-cell inhibition and tumor tolerance (15, 16). In 2020, novel drugs that target the immune checkpoint PD-1/PD-L1 were approved for the treatment of advanced HNSCC (17). More recent studies (18, 19) have shown the association between PD-L1 expression in OPMDs and malignant transformation. Moreover, they suggested that PD-L1 expression in epithelial and subepithelial cells suppresses antitumor immunity and causes malignant transformation in OPMD lesions (18, 20). There are many mechanisms that regulate PD-L1 expression in the tumor microenvironment, and the PI3K-Akt-mTOR pathway is involved in PD-L1 expression. The study reveals the reduced of PD-L1 level in non-small cell lung carcinoma that inhibit mTOR by rapamycin (14). Moreover, in the study of HNSCC cell line demonstrated the mTOR upregulation in the PD-L1 overexpression tumor cell line (21). However, the association between PD-L1 and RPS6 in the malignant transformation of OSCC is still limited, and the association between these biological protein markers and various clinicopathological parameters is inconsistent. Therefore, we evaluated the expression levels of PD-L1 and phosphorylated RPS6 (p-RPS6) in 48 cases of OED and 52 cases of OSCC and determined the correlation of these protein markers with clinicopathological parameters.

## Materials and methods

### Participants and specimens

Formalin-fixed paraffin-embedded (FFPE) tissue blocks of 100 patients, including 52 cases of OSCC and 48 cases of OED, were recruited from archived tissue blocks from 2012 to 2017 at the Department of Oral Pathology, Faculty of Dentistry, Chulalongkorn University, Bangkok, Thailand. Two pathologists (R.C., J.H.) performed the microscopic review of the H&E slides of all cases and established the diagnosis based on the criteria defined in the 2017 WHO classification of head and neck tumors (3). Clinical information, including sex, age, location, duration, greatest dimension, and clinical features, was retrieved from patient medical records. This study was approved by The Human Research Ethics Committee of the Faculty of Dentistry, Chulalongkorn University, Bangkok, Thailand (study code HREC-DCU 2021-006).

### Immunohistochemistry

The FFPE tissue blocks were sliced into three micrometer-thick tissue sections. Immunohistochemistry was performed on the tissue sections according to standard procedure. The slides were placed in xylene and graded alcohol solution for deparaffinization and rehydration. Then, rehydrated slides were submerged into sodium citrate pH 6.0, and antigen retrieval was performed using a microwave heated pressure cooker at 125 °C for 10 min following 30 min of sub-boiling temperature (95–98 °C). The slides were rinsed with water three times and incubated with 3% hydrogen peroxide to eliminate endogenous peroxidase activity. To attenuate nonspecific protein binding, they were incubated in 5% bovine serum albumin for 1 h. They were treated overnight at 4 °C in a humidified chamber with monoclonal rabbit antibodies against PD-L1 (clone 28-8, Abcam, Cambridge, United Kingdom; 1:100) and p-RPS6 (clone 240/244, Cell Signaling Technologies, Beverly, MA, USA; 1:2,000). The amplifier and detector (ab209101, Abcam,) were applied for 30 min, and slides were visualized with diaminobenzidine (Dako North America, Inc., Carpinteria, CA, USA) solution and counterstained with hematoxylin. The tissues of human tonsil and oral squamous cell carcinoma were positive controls for PD-L1 and p-RPS6, respectively. Rabbit isotype-matched monoclonal IgG was replaced with the primary antibody for the negative control.

### Evaluation of immunohistochemical staining

**Figure 1:**
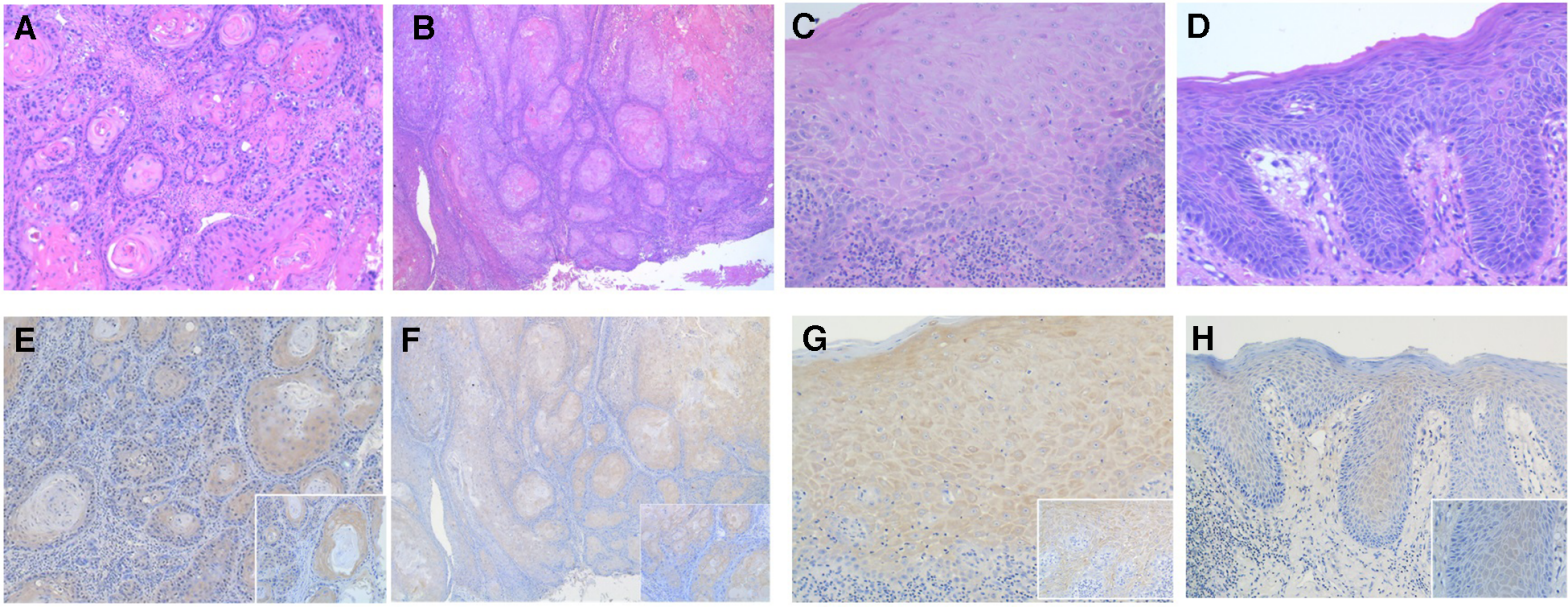
Photomicrographs showing hematoxylin-eosin stanning of (**A,B**) OSCC and (**C,D**) OED. (original magnification: **A** and **B** ×100; **C** and **D** ×200). The PD-L1 immunohistochemical expression in OED and OSCC using PD-L1 specific monoclonal antibody. (**E**) The intensity of PD-L1 immunostaining in OSCC was graded as score 3 and (**F**) score 2 (original magnification: **E** and **F** ×100; inset ×400). (**G**) The intensity of PD-L1 immunostaining in OED was graded as score 3 and (**H**) score 2 (original magnification: **G** and **H** ×200; inset ×400).

**Figure 2:**
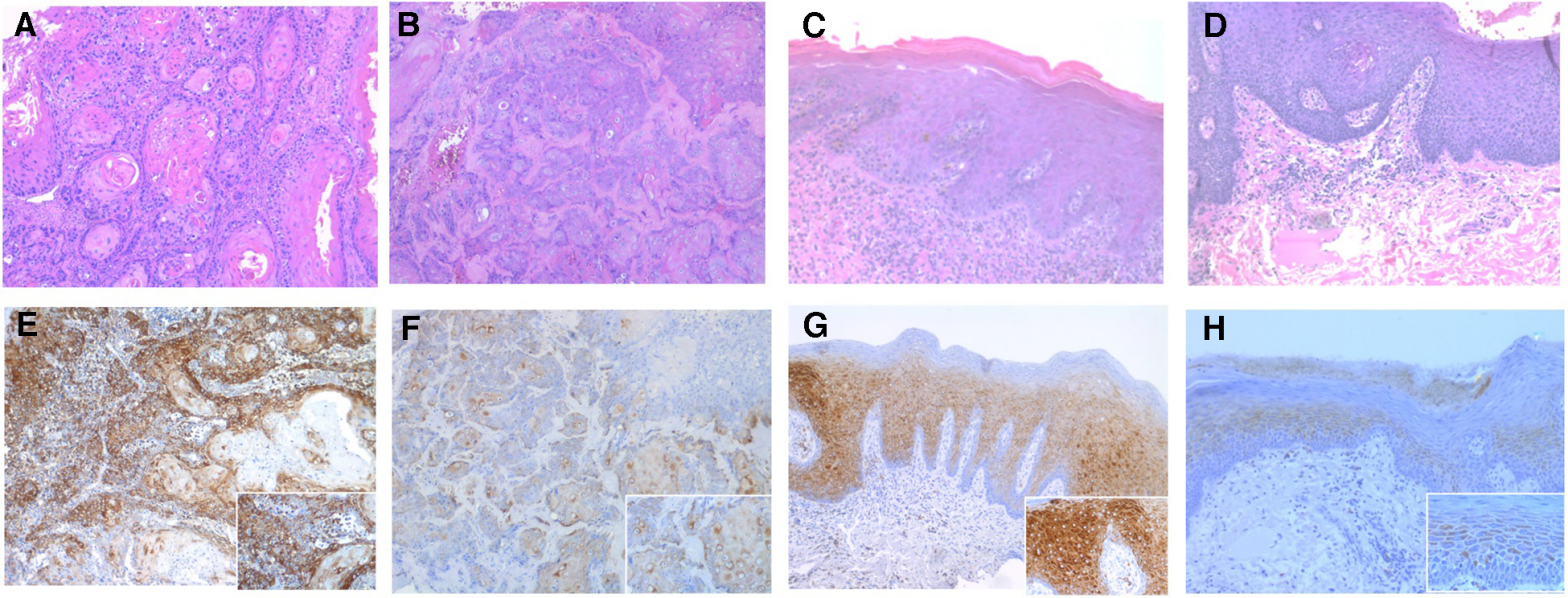
Photomicrographs showing hematoxylin-eosin stanning of (**A,B**) OSCC and (**C,D**) OED. (original magnification: **A–D** ×100). The p-RPS6 immunohistochemical expression in OED and OSCC using p-RPS6 monoclonal antibody. (**E**) The intensity of p-RPS6 immunostaining in OSCC was graded as score 3 and (**F**) score 2, (original magnification: **E** and **F** ×100; inset ×400). (**G**) The intensity of p-RPS6 immunostaining in OED was graded as score 3 and (h) score 2 (original magnification: **G** and **H** ×100; inset ×400).

Cytoplasmic or membranous staining with a golden brown color was considered positive. The PD-L1 and p-RPS6 immunoreactivity were scored based on the intensity and percentage of the cells, according to previous studies (19, 22) with some modification. The percentage and intensity of positive cells was semiquantitatively accessed by examining the entire tissue sections. The percentage of positive cells was scored as follows: 0 (no positive cells), 1 (1%–19% positive cells), 2 (20%–49% positive cells) and 3 (≥50% positive cells). The intensity of positive staining was scored as follows: 0 (negative), 1 (weakly positive), 2 (moderately positive) and 3 (strongly positive) (Figures 1, 2). Two oral pathologists (R.C., J.H.) independently evaluated the entire tissue sections for each case. Discordant cases were resolved by discussion until an agreement was reached.

### Statistical analysis

Statistical analysis was performed using SPSS for Windows version 28.0 software (SPSS Inc., Chicago, IL, USA). The clinical characteristics and immunohistological findings were analyzed using the Kruskal‒Wallis test for differences between multiple groups and the Mann‒Whitney U test for differences between two groups. For continuous variables, the independent sample *t*-test was used to compare the groups. The Spearman test was used to analyze the correlation between PD-L1 and p-RPS6 expression. The chi-square and Fisher's exact test was used to analyze the correlation between PD-L1 positivity and clinicopathologic parameters. The statistical significance was indicated at a *p* value less than 0.05.

## Results

### Clinical and histologic features of OSCC and OED

There were 52 cases of OSCC and 48 cases of OED in this study. The average ages of the OED and OSCC patients were 58 years (range 31–81 years) and 60 years (range 23–83 years), respectively. There were slightly more females than males in both the OED (64% of females) and OSCC (59% of females) groups. The most common location of OED and OSCC was tongue, followed by gingiva and buccal mucosa. The mean lesion or tumor sizes of OED and OSCC were 1.5 cm (range 0.2–4.0 cm) and 2.02 cm (range 0.5–6 cm), respectively. More than half of the OSCC patients showed clinical presentation with painless (59%) and swollen masses (63%). In OED patients, 29 (60%) cases were painless, and 37 (77%) cases were nonhomogeneous surface lesions. According to the histological classification of OSCC, there were 37 cases of well-differentiated and 15 cases of moderately differentiated OSCC. The histological classification of OED was based on the extension of the architectural and cytological dysplastic changes. In all, there are 16 mild epithelial dysplasia, 18 moderate epithelial dysplasia, and 14 severe epithelial dysplasia.

### Correlation between clinical parameters and PD-L1 expression

The clinical characteristics and immunoreactivity for PD-L1 in OED and OSCC are summarized in Tables 1, 2. PD-L1 expression was significantly associated with the greatest diameter of ≥2 cm tumor size in OSCC patients. There was no significant correlation between clinical characteristics of age, sex, location, surface texture, color, pain, paresthesia, and PD-L1 immunoreactivity. The correlation between clinicopathological parameters and p-RPS6 immunoreactivity could not be determined because all cases were positive for p-RPS6.

**Table 1:** Correlation between clinical parameters and PD-L1 expression in OSCC patients.

Continuous variable	*n*	PD-L1			*p* value
		Positive	Negative		
Age (years)	52				0.930
Mean ± SD		58.72 (±13.18)	59.24 (±14.57)		
Range		35–82	23–83		
Lesion size; greatest diameter (CM)	49				0.567
Mean ± SD		2.10 (±1.0)	1.97 (±1.43)		
Range		0.5–5.0	0.5–6.0		
Categorical variables		*n*	PD-L1		
			Positive	Negative	
Gender					0.127
Male		21	14	7	
Female		31	14	17	
Age					0.337
<60		21	13	8	
≥60		31	15	16	
Location					0.268^a^
Buccal mucosa		9	6	3	
Floor of mouth		3	1	2	
Gingiva		14	7	7	
Hard palate		2	2	0	
Lower lip		3	3	0	
Mucobuccal fold		2	1	1	
Retromolar trigone		3	0	3	
Tongue		16	8	8	
Lesion size; greatest diameter (cm)					0.007*
<2 cm		20	6	14	
≥2 cm		29	20	9	
Clinical signs and symptoms					
Swelling					0.198
Swelling, mass		33	20	13	
No swelling		19	8	11	
Pain and paresthesia					0.862
Pain and/or paresthesia		21	11	10	
No pain and/or paresthesia		31	17	14	
Color					0.459^a^
White		2	0	2	
Red		14	9	5	
White and red		12	5	7	
Normal in color/pale pink		6	3	3	
Not identified		18	11	7	
Surface					0.634^a^
Homogeneous/smooth		4	2	2	
Non-homogenous surface		48	26	22	

SD, standard deviation; *n*, number of cases.

^a^ Fisher’s exact test.

*Statistically significant differences (*p* < 0.05).

**Table 2:** Correlation between clinical parameters and PD-L1 immunoreactivity in OED patients.

Continuous variable	*n*	PD-L1			*p* value
		Positive	Negative		
Age (years)	48				0.289
Mean ± SD		59.32 (±12.14)	50.33 (±9.50)		
Range		31–81	41–60		
Lesion size; greatest diameter (CM)	40				
Mean ± SD		1.40 (±1)	2.66 (±1.15)		0.193
Range		0.2–4.0	2.0–4.0		
Clinical diagnosis	48				
Leukoplakia	13				
Lichen planus	11				
Squamous cell carcinoma	11				
Erytholeukoplakia	10				
Non-specific ulcer	3				
Categorical variables		*n*	PD-L1		
			Positive	Negative	
Gender					0.295^a^
Male		17	16	1	
Female		31	26	5	
Age					0.788^a^
<50		10	9	1	
≥50		38	33	5	
Location					0.170^a^
Buccal mucosa		10	6	4	
Floor of mouth		2	2	0	
Gingiva		11	10	1	
Hard palate		6	6	0	
Lower lip		3	3	0	
Tongue		16	15	1	
Lesion size; greatest diameter (cm)					
<2 cm		24	24	0	0.057^a^
≥2 cm		16	13	3	
Clinical signs and symptoms					
Swelling					0.763^a^
Swelling, mass		2	2	0	
No swelling		46	40	6	
Pain and paresthesia					0.554^a^
Pain and/or paresthesia		19	17	2	
No pain and/or paresthesia		29	25	4	
Color					0.147^a^
White		12	12	0	
Red		6	4	2	
White and red		25	22	3	
Not identified		5	4	1	
Surface					0.312^a^
Homogeneous/smooth		9	7	2	
Non-homogenous surface		39	35	4	

SD, standard deviation; n, number of cases.

^a^ Fisher's exact test.

### Frequency of positive PD-L1 and p-RPS6 in OED and OSCC

The positive rates of PD-L1 and p-RPS6 expression in OED and OSCC are summarized in Table 3. The OED was positive for PD-L1 in 42 of 48 cases (87%). Of the positive cases, 16 (33.3%) had mild dysplasia, 17 (35.4%) had moderate dysplasia, and 9 (18.75%) had severe dysplasia. Twenty-eight cases (53.8%) of OSCC were positive for PD-L1. Of the positive cases, there were 19 (35.1%) well-differentiated OSCC cases and 9 (16.6%) moderately differentiated OSCC cases. The immunoreactivity for p-RPS6 was positive in all cases of OSCC and OED.

**Table 3:** Positive rate and average positivity scores for PD-L1 and p-RPS6.

	PD-L1			p-RPS6		
	Number and % positive cases	Positive score		Number and % positive cases	Positive score	
		Mean (SD)	Median (IQR)		Mean (SD)	Median (IQR)
OED	42/48 (87.5%)	2.27 (±1.06)*	3 (1)	48/48 (100%)	2.79 (±0.50)**	3 (0)
OSCC	28/52 (53.8%)	1.37 (±1.37)	1 (3)	52/52 (100%)	2.31 (±0.85)	3 (2)

OED: oral epithelial dysplasia; OSCC: oral squamous cell carcinoma.

*Statistically significant differences (*p* < 0.001) between OED and OSCC.

**Statistically significant differences (*p *= 0.002) between OED and OSC.

The OED cases showed a significantly higher average percentage of cell positive scores of PD-L1 (*p* < 0.001) and p-RPS6 (*p *=* *0.002) than OSCC cases. Moreover, the average intensity scores of PD-L1 in OED (*p *=* *0.002) were higher than those in OSCC cases (Table 4).

**Table 4:** Correlation between PD-L1 and p-RPS6 immunoreactivity in OED and OSCC.

Variable	PD-L1% cell positive	PD-L1 intensity	RPS6% cell positive	RPS6 intensity
PD-L1% cell positive	1.000			
PD-L1 intensity	0.834**	1.000		
RPS6% cell positive	0.319**	0.295**	1.000	
RPS6 intensity	0.286**	0.325**	0.251*	1.000

*p < 0.05. **p < 0.01.

### Comparison of PD-L1 expression between OED and OSCC

The percentage of positive cells and intensity of PD-L1 expression are summarized in Table 5. The OED showed both percent positivity and staining intensity of PD-L1 higher than OSCC; in other words, mild OED demonstrated a higher percentage of cell positivity (*p *=* *0.002) and intensity (*p *=* *0.003) than well- and moderately differentiated OSCC. The percentage of PD-L1-positive cells in moderate OED was significantly higher (*p *=* *0.01) than that in well-differentiated OSCC. Moreover, the intensity of PD-L1 in mild OED (*p *=* *0.018) was significantly higher than that in moderate OED. Regarding intensity, more than half of the OED and OSCC cases showed weak to moderate PD-L1 expression.

**Table 5:** Percentage of cell positivity and intensity of PD-L1 in OED and OSCC cases.

	Number of cases	PD-L1 cell positive					PD-L1 intensity				
		Score 0	Score 1	Score 2	Score 3		Score 0	Score 1	Score 2	Score 3	
OED	48	6 (12.5%)	4 (8.3%)	9 (18.7%)	29 (60.5%)	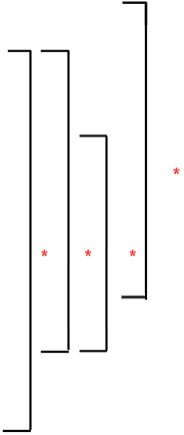	6 (12.5%)	22 (45.8%)	16 (33.3%)	4 (8.4%)	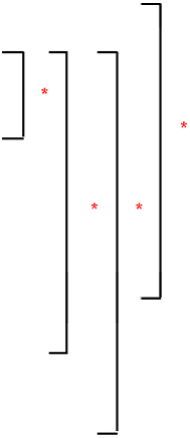
Mild OED	16	0	1 (6.2%)	4 (25%)	11 (68.8%)		0	7 (43.8%)	5 (31.2%)	4 (25%)	
Moderate OED	18	1 (5.6%)	3 (16.7%)	3 (16.7%)	11 (61%)		1 (5.6%)	13 (72.2%)	4 (22.2%)	0	
Severe OED	14	5 (35.7%)	0	2 (14.3%)	7 (50%)		5 (35.7%)	2 (14.3%)	7 (50%)	0	
OSCC	52	24 (46.1%)	3 (5.7%)	7 (13.6%)	18 (34.6%)		24 (46.2%)	15 (28.8%)	10 (19.2%)	3 (5.8%)	
Well-differentiated OSCC	37	18 (48.6%)	2 (5.4%)	4 (10.8%)	13 (35.2%)		18 (48.6%)	9 (24.3%)	7 (18.9%)	3 (8.2%)	
Moderately differentiated OSCC	15	6 (40%)	1 (6.7%)	3 (20%)	5(33.3%)		6 (40%)	6 (40%)	3 (20%)	0	

OED, oral epithelial dysplasia; OSCC, oral squamous cell carcinoma.

*Statistically significant differences (*p* < 0.05).

### Comparison of p-RPS6 expression between OED and OSCC

The percentage of p-RPS6-positive cells and intensity are summarized in Table 6. The percentage of p-RPS6 expression in moderately differentiated OSCC was significantly lower (*p* < 0.001) than that in all OED cases and lower than that in well-differentiated OSCC (*p* < 0.001). In contrast, there were no significant differences in p-RPS6 intensity between OED and OSCC. More than half of OED and OSCC demonstrated a p-RPS6 positive score of 3, and they showed a strong intensity of p-RPS6. However, in the OSCC group, approximately half of the moderately differentiated OSCC cases were positive for a p-RPS6 score of 1.

**Table 6:** Percentage of cell positive and intensity of p-RPS6 in OED and OSCC cases.

	Number of cases	RPS6 cell positive					RPS6 intensity			
		Score 0	Score 1	Score 2	Score 3		Score 0	Score 1	Score 2	Score 3
OED	48	0	2 (4.2%)	6 (12.5%)	40 (83.3%)	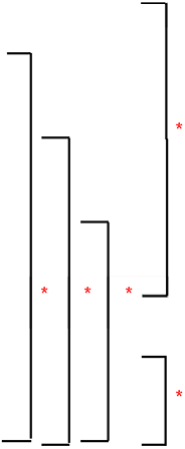	0	0	7 (14.6%)	41 (85.4%)
Mild OED	16	0	1 (6.2%)	2 (12.5%)	13 (81.3%)		0	0	3 (18.7%)	13 (81.3%)
Moderate OED	18	0	1 (5.5%)	2 (11.2%)	15 (83.3%)		0	0	1 (5.6%)	17 (94.4%)
Severe OED	14	0	0	2 (14.3%)	12 (85.7%)		0	0	3 (21.4%)	11 (78.6%)
OSCC	52	0	13 (25%)	10 (19.2%)	29 (55.8%)		0	1 (1.9%)	8 (15.4%)	43 (82.7%)
Well-differentiated OSCC	37	0	6 (16.2%)	4 (11.1%)	27 (72.9%)		0	1 (2.7%)	5 (13.5%)	31 (83.8%)
Moderately differentiated OSCC	15	0	7 (46.7%)	6 (40%)	2 (13.3%)		0	0	3(20%)	12(80%)

OED, oral epithelial dysplasia; OSCC, oral squamous cell carcinoma.

*Statistically significant differences (*p* < 0.001).

### Correlation between PD-L1 and p-RPS6 expression in OED and OSCC

The correlation between PD-L1 and p-RPS6 expression in OED and OSCC patients is shown in Table 4. There were significantly positive correlations between PD-L1 and p-RPS6 expression in OED and OSCC patients (*p* < 0.01).

## Discussion

The checkpoint inhibitor protein PD-L1 is bound to the PD-1 receptor expressed on the cell surface of activated CD8+ T cells in the tumor microenvironment (12–14). The activation of the PD-L1 and PD-1 complex is related to immunosuppression, and promotes tumor progression in various tumors, including renal cell carcinoma, esophageal cancer, stomach cancer, melanoma, and HNSCC (17, 23–25). In recent studies, the level of PD-L1 expression was detected in OSCC than in OED and showed a positive correlation with the advanced histopathologic grades of OSCC (18–20, 24). In 2020, the systematic review and meta-analysis reveals the lower tendency of PD-L1 expression in precancerous lesions than in OSCC without reaching statistical significance (24). However, our study found higher expression of PD-L1 in OED than in OSCC (*p *<* *0.001). In addition, the intensity and percentage of cell positive for PD-L1 were higher in mild OED than in well- and moderately differentiated OSCC. These finding were consistent with Malaspina et al. (26), who demonstrated the higher tendency of PD-L1 expression in precancerous lesion compared to OSCC. Furthermore, the study in oral leukoplakia in 2017 (27), showed that all cases of leukoplakia are positive for PD-L1. In aspects of PD-L1 expression in OSCC, Lenouvel et al. (17) reported that PD-L1 overexpression in OSCC varies between 18% and 96%. The heterogeneity of PD-L1 expression in HNSCC can be influenced by several factors including specimen storage duration, antibody clone types, and tissue section nature (28, 29). Karpathiou's study (28) indicates that longer storage time leads to a reduction in PD-L1 positive cells. Additionally, studies have shown differences in interobserver reliability when comparing whole specimens with microarrays in the PD-L1 evaluation. This could be due to the representativeness of the tissue sections or differences in tissue processing. Variation in platforms used for assessing PD-L1 expression, such as different immunohistochemical staining systems or digital image analysis tools, can lead to divergent interpretations. This might be due to variations in sensitivity, specificity, or image analysis algorithms of these platforms. At present, there is no consensus on a standardized scoring method for PD-L1. Further research with a standardized scoring method is needed to clarify its role in OSCC (30).

The phosphorylation of RPS6 is a significant final event in the Akt/mTOR pathway (5, 8). The mTOR pathway is activated by many cellular signals, including growth factors, hormones, and stress. The activated Akt/mTOR pathway is a critical mediator of cell survival and cell proliferation and is a common pathway in the pathogenesis of OSCC (5, 8, 26). In 2016, the study showed the higher expression of phosphorylated Akt, mTOR in OED and OSCC compared with normal mucosa. However, the expression of S6 kinase protein is no different between normal mucosa, OED and OSCC (8). The essential role of p-RPS6 in HNSCC was firstly described by Amornphimoltham and colleagues (31), in 2005. The authors demonstrated the reduction of RPS6 levels in HNSCC cell lines treated with mTOR inhibitors. In the present study demonstrated that all cases of OED and OSCC were positive for p-RPS6, and the percentage of cells positive for p-RPS6 in OED was significantly higher (*p *=* *0.002) than that in OSCC. This result is in concordance with a previous study on Thai patients. The authors found that the immunohistochemical reaction for p-RPS6 is observed in all cases of OED and 88.8% of OSCC (5). The concordance of studies on the expression of p-RPS6 in OED and OSCC suggests the involvement of the deregulated Akt/mTOR pathway in the early events of tumor progression and the pathogenesis of OSCC. The varying immunohistochemical reaction for Akt, mTOR, S6 kinase and RPS6 level in OED and OSCC is observed, and few studies assessed p4EBP1 expression. A future study that reveals both p-RPS6 and p4EBP1 in OED and OSCC may reveal the association of Akt/mTOR pathways and carcinogenesis of OED and OSCC.

The association between the Akt/mTOR pathways and PD-L1 expression has been revealed in several studies (14, 32, 33). The study of inhibiting mTOR in non-small cell lung carcinoma shows a reduced PD-L1 expression level. Moreover, a recent study found that overexpression of PD-L1 in tumors that lost the expression of PTEN resulted in the upregulation of the Akt/mTOR pathway (34). Our findings demonstrated that PD-L1 and p-RPS6 expression was significantly higher in OED patients than in OSCC patients (*p* < 0.05). PD-L1 expression in the OED was decreased in the advanced histological grades of OED, whereas the expression level of p-RPS6 did not differ between the three grades of the OED. In OSCC, PD-L1 Expression was slightly increased in moderately differentiated OSCC compared to well-differentiated OSCC. In contrast, p-RPS6 showed a significantly reduced expression level in the moderately differentiated OSCC patients (*p* < 0.001). Our studies demonstrated a significant positive correlation between PD-L1 and p-RPS6 expression in OED and OSCC (*p* < 0.05) (Table 6). In a study by Zheng et al. (21), PD-L1 overexpression in HNSCC cell lines resulted in higher levels of p-RPS6 and p-Akt compared with the PD-L1 negative HNSCC group. Based on previous findings, the higher PD-L1 and p-RPS6 expression in the early stage of OED than in OSCC may imply that the PD-L1 and Akt/mTOR pathways are activated in the early development of OSCC. The role of PD-L1 may associated in the early stages of tumor progression.

Our findings revealed that the expression of PD-L1 was not correlated with clinical characteristics, including sex, age, location, greatest diameter, or clinical features. PD-L1 expression in OED and OSCC was slightly higher in males than in females, but it did not show a significant difference in our study. The association between the expression of PD-L1 and sex is inconsistent in the literatures. Lin et al. (35). reported significantly higher intensity for PD-L1 cytoplasmic staining in males than in females; in contrast, Satgunaseelan et al. (36) found that the expression of PD-L1 was significantly higher in females. The affected sites of OED, including the lateral tongue and floor of the mouth, and a lesion diameter greater than 2 cm are associated with the high potency of malignant transformation of OED (37). Our study demonstrated that PD-L1 and p-RPS6 expression was not correlated with the locations and greatest diameter in OED. Tumor localization and PD-L1 expression vary (17). In recent studies, OSCC arising at the mandibular structures and tongue showed significantly higher expression than OSCC arising at the maxilla or soft palate. Satgunaaseelan et al. (36) showed that PD-L1-positive OSCC was localized in the lingual and buccal mucosa compared to the gingiva and floor of the mouth (*p *=* *0.05). Our study revealed that PD-L1 expression was significantly related to a tumor size greater than 2 cm in OSCC patients. Kouketsu et al. (19) reported a significant correlation of tumor size and PD-L1 expression, and PD-L1 was higher in advanced TNM staging. Few studies have shown discordant results on the relationship between tumor size and PD-L1 expression, and some studies have demonstrated no correlation (35, 38).

The role of PD-L1 expression in OSCC has significant implications for the treatment of this aggressive cancer type. Traditionally, the primary approach modalities for OSCC included surgical excision with or without radiotherapy. Recent advancements in cancer treatment have introduced immunotherapy, specifically anti-PD-1 and anti-PD-L1 therapies, as promising options for subset of OSCC patients. These therapies represent a paradigm shift from traditional treatments, focusing instead on modulating the body's immune response to cancer (39–41). Anti-PD-1 and anti-PD-L1 therapies work by inhibiting the PD-1/PD-L1 pathway. PD-L1, expressed on tumor cells, interacts with PD-1 on T-cells, leading to the suppression of the immune response against the tumor. By blocking this interaction, these therapies can reactivate T-cells, allowing them to effectively target and destroy cancer cells (39). Although some studies showed the mutations and immunological factors could predict resistance and recurrence in the patients treated with neoadjuvant anti-PD-1 therapy for OSCC (40). These therapies represent a significant advancement in OSCC treatment, but also requiring careful consideration of the unique aspects of each patient's cancer.

In conclusion, our findings of a higher percentage of cells positive for PD-L1 and p-RPS6 in OED compared to OSCC suggest that these molecules could contribute a significant role in the early stages of oral tumorigenesis. This could imply that changes in these pathways are early events in the development of OSCC. Moreover, the concordant expression of p-RPS6 and PD-L1 may represent the association of the Akt/mTOR pathway and PD-L1-regulated immunoediting in OED and OSCC. The study of the association of the Akt/mTOR pathway and PD-L1 is still limited, and many biological mechanisms control the expression of PD-L1. Understanding the biological mechanisms that control PD-L1 expression is complex and requires more in-depth research.

## Data Availability

The raw data supporting the conclusions of this article will be made available by the authors, without undue reservation.
